# PDE3A as a Therapeutic Target for the Modulation of Compartmentalised Cyclic Nucleotide-Dependent Signalling

**DOI:** 10.3390/cells14110771

**Published:** 2025-05-23

**Authors:** Swaroop Ranjan Pati, Anastasiia Sholokh, Enno Klussmann

**Affiliations:** 1 Max-Delbrück-Center for Molecular Medicine in the Helmholtz Association (MDC), 13125 Berlin, Germany; swaroopranjan.pati@mdc-berlin.de (S.R.P.); anastasiia.sholokh@mdc-berlin.de (A.S.); 2 Faculty of Biology, Chemistry, Pharmacy, Freie Universität Berlin, 14195 Berlin, Germany; 3 DZHK (German Centre for Cardiovascular Research), Partner Site, 10785 Berlin, Germany

**Keywords:** phosphodiesterase 3A, cAMP, compartmentalisation

## Abstract

Phosphodiesterase 3A (PDE3A) hydrolyses cAMP, adjusting cAMP signalling pathways with temporal and spatial accuracy. PDE3A contributes to the control of cAMP in several cellular compartments, including the plasma membrane, the cytosol, or membrane-limited organelles such as the nucleus and the sarcoplasmic reticulum. Through this ability and its expression in various cell types, it regulates a variety of cellular processes like contractility of muscle cells, gene expression, differentiation and proliferation. Dysregulated cAMP signalling causes or is associated with diseases. The therapeutic potential of PDE3A is, however, limited by the lack of specific modulators. Emerging approaches to targeting PDE3A centre on specifically addressing its catalytic domain or its cellular localisation. This review highlights the growing knowledge of PDE3A’s functions in cellular signalling and therapeutic opportunities, opening the door to more fully utilise its potential for the treatment of disease.

## 1. Introduction

**Figure 1:**
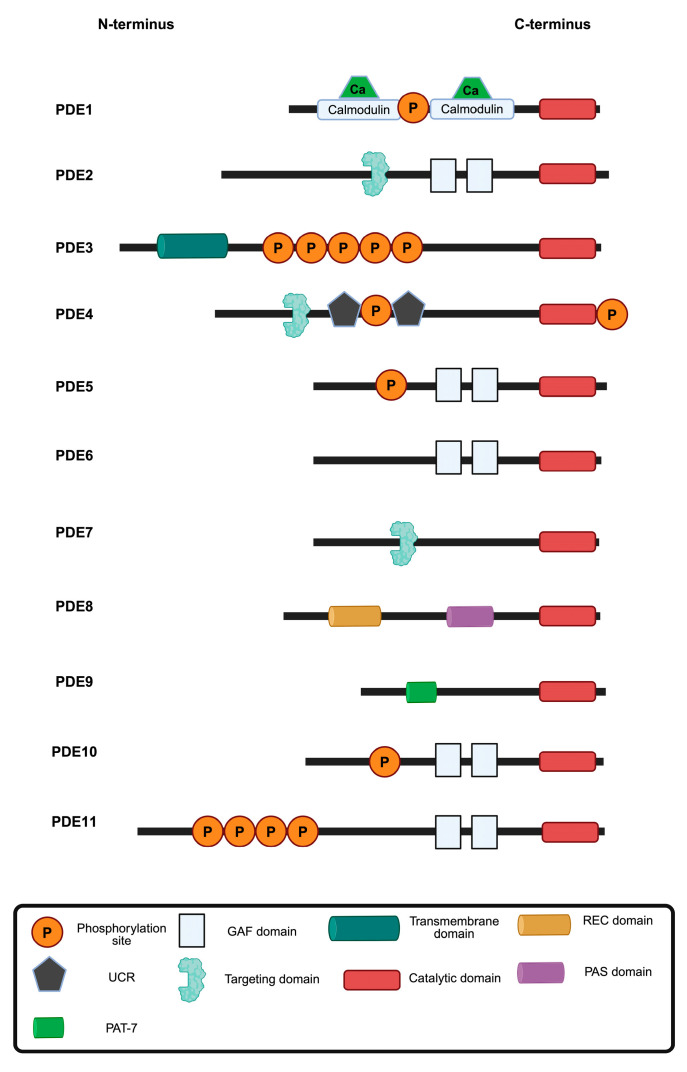
Overview of PDE families 1–11. PDEs hydrolyse cAMP, cGMP or both (dual-specificity; Table 1). Each PDE isoform contains a conserved catalytic domain (red) but exhibits diversity in regulatory domains, localisation signals and post-translational modifications. Calmodulin-binding domains bind Ca^2+^/calmodulin to increase activity. GAF (cGMP-binding ubiquitous motif) domains function as regulatory domains that bind cyclic nucleotides to modulate activity, sense cellular signals and influence localisation. Transmembrane domains mediate membrane localisation. UCR (upstream conversed region) domains mediate dimerization and regulatory interactions. Targeting domains direct cellular localisation and protein interactions. The REC (Signal regulatory) and PAS (PerARNT-Sim) domains serve as sensors of cellular stimuli. PAT-7 (7-residue nuclear localisation signal) may influence tissue-specific expression or protein interactions. The positioning and the specific domains of each PDE contribute to the distinct regulatory properties and cellular functions of each PDE. Adapted from Fu et al. [6]. Figure prepared with BioRender.com.

Cyclic nucleotides (cNTs), such as cyclic adenosine monophosphate (cAMP) and cyclic guanosine monophosphate (cGMP), initiate cellular signalling processes that direct vital functions, e.g., muscle cell contractility, cell differentiation and proliferation or metabolic processes. cNTs are ubiquitous and the specificity in their coordination of cellular processes depends on their compartmentalisation. Phosphodiesterases (PDEs) play a critical role in organising this compartmentalisation by terminating cNT signalling through hydrolysis to 5′-monophosphate forms [1,2,3,4,5,6,7]. They either directly associate with a cellular compartment, such as the nucleus or the plasma membrane or they are tethered by anchoring proteins, such as A-kinase anchoring proteins (AKAPs) [8,9,10,11]. PDEs are constitutively active and thereby maintain a low level of cNTs in their vicinity, limiting their diffusion. If the stimulation of cells with agonists induces cNT generation above threshold concentrations, cNTs hydrolysis by PDEs is overcome and cNTs can initiate downstream signalling. PDEs are encoded by 21 genes that give rise to 11 families (PDE1-11) of PDEs with more than 100 enzyme isoforms (Figure 1). The PDE4, PDE7 and PDE8 family enzymes specifically hydrolyse cAMP, the PDE5, PDE6 and PDE9 families are cGMP-specific, while the PDE1, PDE2, PDE3, PDE10 and PDE11 family members hydrolyse both cAMP and cGMP (Table 1) [12,13,14,15]. The individual enzymes differ in structure, regulation, location and pharmacological properties. The different PDE families and crosstalk between members of the different families have recently been discussed in detail in two excellent review articles [6,7].

Based on recent elucidations of novel aspects of PDE3A functions and its therapeutic potential, this review focusses on how PDE3A’s compartment-specific regulation of cAMP is essential for preserving cellular and physiological balance. The therapeutic potential of the enzyme is highlighted and the need for next-generation strategies to overcome the limitations of traditional treatments directed non-selectively at the PDE3 family is pointed out.

## 2. Structural and Functional Insights into PDE3A

### 2.1. PDE3A Structure and Isoforms

**Figure 2:**
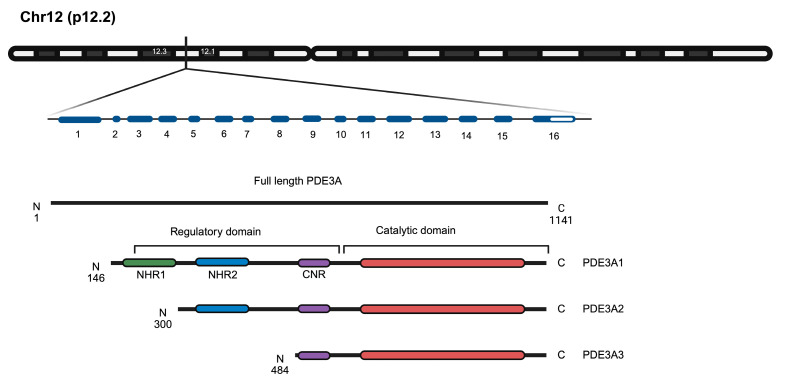
Schematic representation of the PDE3A gene and the encoded PDE3A1, PDE3A2 and PDE3A3 enzyme isoforms. The chromosomal location of the gene (p12.2) and the exons (blue) are indicated. The full-length PDE3A protein (1141 amino acids) and the isoforms are depicted and the regulatory and catalytic domains are highlighted. The three PDE3A isoforms show variations in their N-terminal regions while maintaining identical catalytic domains (red). NHR, N-terminal hydrophobic regions 1 and 2 (green and blue, respectively); CNR, common N-terminal region (purple) [6]. Figure prepared with BioRender.com.

PDE3A, together with PDE3B, forms the PDE3 family. The single PDE3A gene on chromosome 12p12.2 gives rise to three PDE3A enzyme isoforms, PDE3A1, A2 and A3 through alternative splicing (Figure 2).

The catalytic domains (amino acids 665–1141 in PDE3A1) are identical in all three PDE3A isoforms. The isoforms differ in the lengths of their N termini (Figure 2). The N-terminal hydrophobic regions (NHR) 1 and 2 direct PDE3A1 and 2 to lipid membranes, while an NHR is missing from PDE3A3, which therefore is found cytosolic. However, the localisation of PDE3A is not fully understood. PDE3A1 and A2 despite their NHRs are both membrane-associated and cytosolic, e.g., in HEK293 cells [16,17].

**Figure 3:**
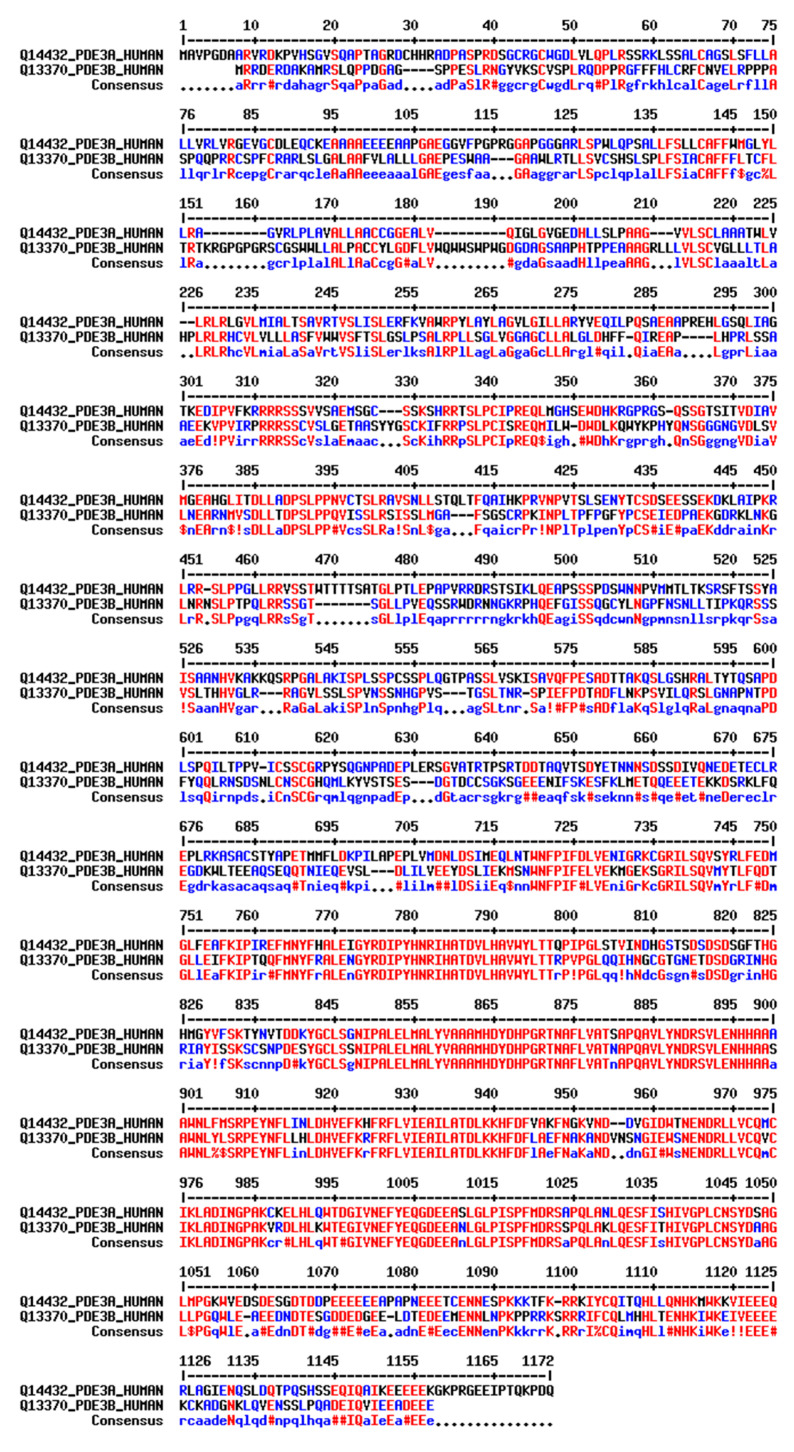
Sequence alignment of full-length human PDE3A and PDE3B. Identical amino acids are in red, while similar residues are shown in blue. Gaps in the alignment are indicated by dashes (-), representing regions of sequence divergence. The regulatory domain extends up to residue 664, while the catalytic domain starts after residue 664. The numbering is based on the PDE3A1 sequence. The consensus sequence is displayed at the bottom. The sequence alignment was adapted from PDE3A and PDE3B UniProt sequences (IDs Q14432 and Q13370, respectively) using multalin (http://multalin.toulouse.inra.fr/multalin/) (accessed on 28 March 2000) [19].

PDE3B is encoded by a single gene on chromosome 11p15.1 and gives rise to a single isoform. While the N termini of PDE3A and PDE3B share 35% sequence identity, 47% sequences similarity and 28% gaps, their catalytic domains display 64% sequence identity, 78% sequence similarity and 14% gaps (Figure 3). Both PDE3A and PDE3B are competitively inhibited by cGMP and hydrolyse cAMP. The affinity for cAMP and cGMP is similar (K_m_ = 0.1–0.8 μmol/L); the V_max_ for cAMP is 4–10 times higher than for cGMP. The inhibition constant (K_i_) of cGMP is around 0.06 μM [18].

### 2.2. Phosphorylations Control PDE3A Function and Location

**Figure 4:**
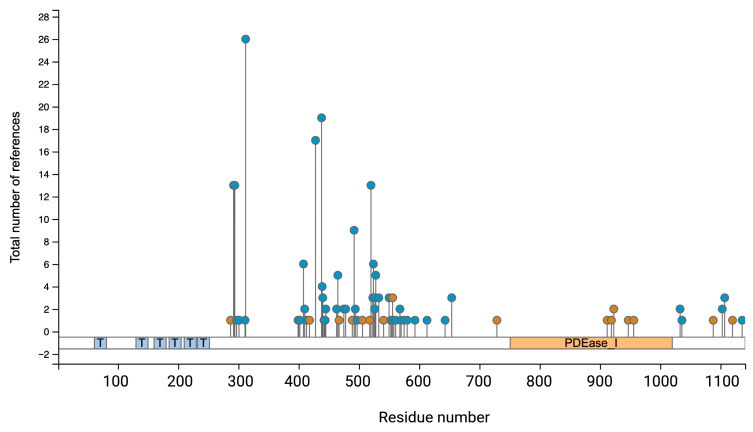
Post-translational modifications (PTM) of human PDE3A. The diagram illustrates the distribution of phosphorylation (blue circles) and ubiquitination (brown circles) sites along the PDE3A1 amino acid sequence, annotated by the residue number on the x-axis, and the sky blue boxes with “T” denote transmembrane helixes positions. The y-axis denotes numbers of references reporting each PTM site, based on aggregated phospho- and ubiquitylome datasets. The PDE3A catalytic domain (PDEase) is highlighted in orange. Multiple serine, threonine, and tyrosine residues across the protein may be phosphorylated; they appear particularly clustered between residues 300 and 600. Ubiquitination sites are dispersed but as opposed to phosphorylation sites are also located in the catalytic domain. This figure was generated using curated data from the PhosphoSitePlus database (www.phosphosite.org).

The PDE3A isoforms contain several phosphorylation sites N- and C-terminally from their catalytic domain [20,21,22] (Figure 4). The phosphorylations of serine (Ser)290–292, Ser312, Ser428, Ser438, Ser465, Ser492, Ser520, Ser524, Ser528, T568 and S654 have been identified experimentally [23,24] and by computational predictions (www.phosphosite.org) but their function is largely unclear (Figure 4, Table 2). Understanding such post-translational modifications is essential for clarifying PDE3A’s function in cellular signalling and their potential as a therapeutic target.

The phosphorylation pattern of PDE3A changes in response to extracellular cues, such as agonists that stimulate G protein-coupled receptors, which cause activation of stimulatory G proteins (G_S_). Activated G_S_ stimulates adenylyl cyclases to synthesise cAMP. The cAMP activates its effectors, the main effector protein kinase A (PKA) [25], but also exchange proteins activated by cAMP (Epac1 and 2) [26], Popeye domain-containing (Popdc) proteins [27] and cyclic nucleotide-gated ion (CNGC) and hyperpolarization-activated cyclic nucleotide-gated (HCNC) channels [28]. Agonists stimulating other receptors, such as those coupled to the G protein Gq, lead to activation of other pathways and kinases, e.g., protein kinase C (PKC) in the case of Gq activation.

Several kinases, including PKA, PKC, protein kinase G (PKG) and protein kinase B (PKB/Akt) phosphorylate PDE3A (Table 2). Akt/PKB phosphorylates it at the Ser290–292 cluster. Akt-dependent PDE3A phosphorylation leads to its activation and reduces the intracellular cAMP level, which allows oocytes to resume the meiosis process; PDE3A unphosphorylated at the cluster results in meiotic arrest in oocytes [29].

**Table 2:** PDE3A phosphorylation sites, the kinase phosphorylating them and the function. The numbering of the phosphorylation sites relates to the amino acid sequence of PDE3A1.

Phosphorylation Site	Kinase/Mediator	Cellular Function/Outcome	Citation
Ser290–292	Akt (PKB)	Phosphorylation increases PDE3A activity and regulates oocyte maturation in response to PI3K signalling.	[29]
Ser312	PKA	Enhances catalytic activity of PDE3A; involved in feedback regulation of cAMP levels. Induces binding of 14-3-3 proteins.	[30,31,32]
Ser428	PKC	Facilitates binding to 14-3-3 proteins; may influence PDE3A localization and stability.	[30,31,32]
Ser438	PKC	Promotes 14-3-3 binding; associated with increased PDE3A activity in [30,31,32] platelets.	[30,31,32]
Ser465	PKC	Associated with 14-3-3 interaction and activation during platelet activation.	[30,31,32]
Ser492	PKC	Correlates with enhanced PDE3A activity; 14-3-3 binding during platelet activation.	[30,31,32]
Ser520	Unknown	Unclear	www.phosphosite.org
Ser524	Unknown	Unclear	www.phosphosite.org
Ser528	Unknown	Unclear	www.phosphosite.org
Ser654	PKG	Regulatory role and mediates proteasomal degradation of PDE3A.	[33]

Exposure of HEK293 cells to the β-adrenergic receptor (βAR) agonist isoproterenol and hence activation of PKA led to phosphorylation of PDE3A1 at S312. The phosphorylation therefore creates a negative feedback loop that lowers intracellular cAMP and PKA activity [31]. Activation of PKC with phorbol 12-myristate 13-acetate (PMA) led to phosphorylation of PDE3A2 at S428 [31,32]. The phosphorylation of PDE3A1 at S312 or S428 did not affect its activity. However, the phosphorylation of S428 stimulated PDE3A2 activity [31]. The increased S428 phosphorylation and PDE3A2 activity was consistent with the observation that hyperactivity-causing PDE3A mutations [17,20] were associated with increased S428 phosphorylation in HeLa and HEK293 cells [16,34]. Both phosphorylation of S312 and S428 induced 14-3-3 binding [31].

When platelets are activated by agonists like thrombin or the peptide SFLLRN, a PAR-1 agonist, PKC phosphorylates Ser438, Ser465, and Ser492 and 14-3-3 binding to PDE3A increases. The interaction is associated with increased PDE3A activity [30,32]. PKC is predominately activated through Gq and downstream Ca^2+^ and diacylglycerol (DAG) but not cAMP. Thus, crosstalk via PKC can increase PDE3A-mediated cAMP hydrolysis and thereby terminate cAMP signalling and facilitating the return to baseline cAMP levels.

PKG phosphorylates PDE3A at Ser654 in endothelial cell which increases the cAMP level by disrupting the interaction of PDE3A with HSP90 and leads to its ubiquitin-mediated proteasomal degradation [33]. Prediction by PhosphoSite (www.phosphosite.org) has detected several ubiquitylation sites on PDE3A (Figure 4) which could facilitate the ubiquitin ligases-mediated ubiquitination and proteasomal degradation of PDE3A.

**Figure 5:**
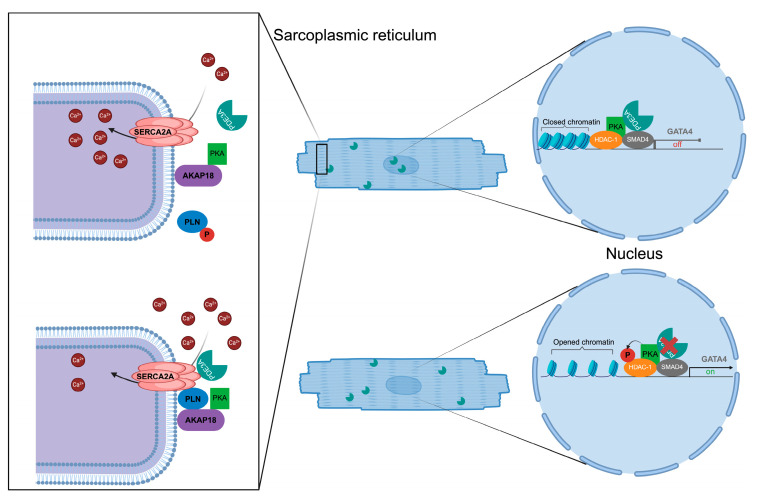
PDE3A compartments in cardiomyocytes. Sarcoplasmic reticulum Ca^2+^ ATPase (SERCA) 2a-mediated Ca^2+^ reuptake from the cytosol into the sarcoplasmic reticulum of cardiomyocytes. Left: protein complex organised by AKAP18 comprising SERCA2a, phospholamban (PLN), protein kinase A (PKA) and PDE3A under basal conditions when SERCA2a is inhibited by binding of PLN. Elevation of cAMP causes PKA activation, PKA phosphorylation of PLN, dissociation of PLN and PDE3A and activation of SERCA2a. Activated SERCA2a pumps Ca^2+^ into the SR during diastole [6]. Right: In the nucleus, PDE3A2 hydrolyses cAMP and thereby controls PKA activity and hypertrophic gene expression through a histone deacetylase 1 (HDAC-1)-SMAD family member 4 (SMAD4) complex. For details see text. Figure prepared with BioRender.com.

PDE3A’s subcellular location influences its phosphorylation, which in turn may have an impact on its localisation. PKA, PDE3A and their substrates are brought together by compartment-specific signalling platforms, such as those involving AKAPs, which spatially limits phosphorylation events [35]. For example, in case of cardiomyocytes AKAP18 interacts with PDE3A near the sarcoplasmic reticulum (SR) at the SR Ca^2+^ ATPase 2a (SERCA2a) complex (see below Figure 5). Phosphorylation of PDE3A1 by PKA enhances its association with this complex. PDE3A phosphorylation and activity are negatively impacted by disruption of AKAP-PKA interactions [36,37]. In human platelets, AKAP7 forms a complex with PDE3A and PKA in the cytosol. In response to prostacyclin, PGI_2_, this complex alters cAMP levels in the cytosol, which impacts platelet activation [22].

Apart from AKAPs, PDE3A interacts with the guanine nucleotide exchange factors BIG1 and BIG2, which are involved in vesicular trafficking in HeLa cells. This interaction positions PDE3A as a potential regulator of ADP-ribosylation factor 1 (ARF1) activity and membrane trafficking, as it co-localises with BIG1/2 at the Golgi and endosomal membranes. Their scaffold-like properties suggest that BIG1 and BIG2 facilitate the anchoring of PDE3A to specific subcellular compartments. However, the phosphorylation status of PDE3A in these compartments within HeLa cells remains unknown [38]. Additionally, PDE3A’s phosphorylation-induced conformational changes may modify how it interacts with membranes or anchoring partners, changing its spatiotemporal regulation of cAMP signalling [39].

Overall, it appears as if the phosphorylations mainly regulate PDE3A activity and interactions with scaffolding proteins like AKAPs, BIG1 and BIG2 which determine the cellular compartmentalisation of PDE3A. The ubiquitination regulates proteasomal degradation of PDE3A [33].

### 2.3. PDE3A Expression Pattern: Distinct PDE3A Compartments in the Same Cell

The expression pattern of PDE3A and B differ (Table 3 and Table 4). PDE3A is expressed in various cell types (Table 4) and located in several cellular compartments, including the plasma membrane, the cytosol and membrane-limited organelles such as the nucleus and the SR; PDE3A controls the amplitude and duration of cAMP signalling at these locations [40,41].

PDE3A in cardiomyocytes exemplifies how a single enzyme controls cAMP in distinct cellular compartments, the plasma membrane (sarcolemma), the cytoplasm (sarcoplasm), nucleus and the SR. PDE3A plays a critical role in regulating cardiac contractility. Contraction of cardiomyocytes, which mediates cardiac contraction, is induced by the elevation of cytosolic Ca^2+^. Ca^2+^ influx via L-type voltage-gated Ca^2+^ channels (Ca_V_1.2; LTCC) enhances cytosolic Ca^2+^ directly and indirectly through Ca^2+^-induced Ca^2+^ release from the SR, the intracellular Ca^2+^ store, through opening of SR-located ryanodine type 2 receptors (RyR_2_). Relaxation (diastole) is induced by the removal of Ca^2+^ from the cytosol through reuptake into the SR by SERCA2a and efflux from the cells through ion transporters. The stress hormone adrenaline and the neurotransmitter noradrenaline greatly enhance Ca_V_1.2 currents and thereby cardiac contractility via activation of βARs, β_1_AR and β_2_AR, at the plasma membrane and downstream signalling in defined cellular compartments. Increased βAR-Ca_V_1.2 signalling is a fundamental physiological process underlying the “fight-or-flight” response. βAR activation causes a rise in cAMP, triggering the activation of PKA [67,68,69,70,71]. By phosphorylating a number of downstream targets, PKA enhances Ca^2+^ cycling and cardiac contractility, causing positive inotropic, lusitropic, dromotropic and chronotropic responses that adjust the cardiac output to meet increased physiological demands.

PDE3, and also PDE4, are part of βAR-related signalosomes in cardiomyocytes, and both contribute to the termination of βAR signalling. Microscopic analyses based on biosensors and Förster resonance energy transfer (FRET) revealed that PDE3 is located in non-lipid raft sarcolemma regions and contributes to regulating βAR signalling. However, it is not clear whether this involves PDE3A or/and 3B [72].

Phospholamban (PLN) inhibits SERCA2a and thereby lowers Ca^2+^ reuptake into the SR during diastole (Figure 5). The phosphorylation of PLN is induced by PKA in response to βAR stimulation at Ser16, relieving its inhibitory action on SERCA2a. This SERCA2a activation enhances Ca^2+^ sequestration into the SR. PKA and PLN together with PDE3A are tethered to the SR membrane in the vicinity of SERCA2a by AKAP18 (Figure 5) [37,73]. PDE3A at the complex hydrolyses cAMP to limit PKA activity locally and thus the phosphorylation of PLN [74,75,76,77,78,79]. Mutations of the PDE3A gene causing hyperactivity of PDE3A reduced the phosphorylation of PLN at Ser16 in rat hearts, confirming the involvement of PDE3A in the control of local cAMP, PKA activity, PLN Ser16 phosphorylation and thus of Ca^2+^ reuptake into the SR during diastole [17]. However, it is not only the PDE3A-mediated local cAMP hydrolysis that is relevant for controlling SERCA2a activity. PDE3A directly interacts with SERCA2a and disruption of the interaction with peptides increased SERCA2a activity in cardiomyocytes isolated from mice [80]. However, it is not clear to what extent the local PDE3A-mediated cAMP hydrolysis, and thus low PKA activity and decreased Ser16 PLN phosphorylation, and the mere direct interaction of PDE3A with SERCA2a control SERCA2a activity.

A phosphoproteomics-based approach in combination with the use of nucleus-directed cAMP-FRET sensors provided insight into the function of PDE3A in the nucleus [41]. The data revealed that active PDE3A2 at a nuclear complex comprising SMAD family member 4 (SMAD4) and histone deacetylase 1 (HDAC-1) locally hydrolyses cAMP and thus maintains PKA inactive. Inhibition of PDE3A, i.e., non-selective inhibition with cilostamide, or displacement of PDE3A2 from the complex, resulted in locally increased cAMP, local PKA activation and phosphorylation of HDAC-1. HDAC-1 deacetylates histones, repressing expression of prohypertrophic genes. The PKA phosphorylation inhibits its deacetylase activity. As a consequence, prohypertrophic gene transcription enhanced, promoting cardiomyocyte hypertrophy [41].

Altogether, in cardiomyocytes PDE3A contributes to limiting the local cAMP level at various locations to prevent excessive PKA activation, which in turn protects from Ca^2+^ overload in the cytosol and maladaptive cardiomyocyte and cardiac remodelling and eventually from heart failure, which is promoted by catecholamine overstimulation of βARs. The role of cytosolic PDE3A is not clear. One role is most likely to provide the storage compartment for the pool of PDE3A that dissociates from SERCA2a during the SERCA2a activation process.

Similarly to cardiomyocytes, PDE3A resides in distinct subcellular compartments in vascular smooth muscle cells (VSMCs), where it is involved in the regulation of excitation-contraction coupling and cell proliferation. In VSMCs, activation of GPCRs by vasoconstrictors (e.g., angiotensin II, norepinephrine) activates phospholipase C and subsequent production of inositol trisphosphate (IP3), which then induces Ca^2+^ release from the SR. Membrane depolarisation opens LTCCs, facilitating Ca^2+^ influx. The rise in intracellular Ca^2+^ allows Ca^2+^-calmodulin to activate myosin light-chain kinase (MLCK), which phosphorylates myosin light-chain (MLC) at Ser19. Phosphorylated MLC enables cross-bridge cycling between actin and myosin, enabling contraction. VSMCs relax when cytosolic Ca^2+^ decreases, MLCK is inactivated, and MLC is dephosphorylated by myosin light-chain phosphatase (MLCP). PKA promotes relaxation both by inhibiting MLCK and by phosphorylating the MLCP targeting subunit (MYPT1), thereby enhancing MLCP activity [81].

In this context, PDE3A plays a crucial role in promoting VSMCs relaxation via increasing cAMP levels, which is essential for PKA activation and PKA-mediated phosphorylation events leading to vasodilation. Hyperactive PDE3A has been shown to reduce cAMP levels in VSMCs, impairing their ability to relax and contributing to increased vascular tone. This mechanism underlies the development of hypertension in hypertension with brachydactyly [16]. PDE3A expression was detected in the cytosolic fractions of cultured aortic VSMC homogenates [48]. Specifically, PDE3A1 is most likely associated with SERCA2 at the endoplasmic reticulum of VSMCs, similarly to findings in cardiomyocytes; however, direct evidence is still lacking.

In addition to its involvement in controlling contractility, PDE3A is also involved in cell cycle control in VSMCs. PDE3A contributes to cell cycle transitions, especially the G1/S checkpoint, by affecting the phosphorylation of retinoblastoma protein (Rb) and other proteins in the nucleus linked to the cell cycle, including cyclin-dependent kinases (CDKs) [82]. Deletion of PDE3A suppressed the proliferation of cultured murine VSMCs via dysregulation of PKA and MAPK signalling, resulting in cell cycle arrest at G0–G1 stage. A similar phenomenon was observed in oocytes, where PDE3A deletion led to cell cycle arrest at the G2/M stage.

PDE3A is expressed in platelets, where it modulates platelet aggregation by regulating intracellular cAMP levels. Since elevated cAMP inhibits platelet activation, PDE3A-mediated cAMP hydrolysis promotes aggregation by lowering cAMP concentrations (see Section 2.2). PDE3A is distributed across different subcellular compartments in platelets. The majority of its enzymatic activity is attributed to the cytosolic fraction, primarily by PDE3A2, and potentially PDE3A3. Although PDE3A1 has been detected in membrane-associated fractions, its contribution to total PDE3A activity in platelets appears to be minimal. Importantly, cytosolic PDE3A forms part of a signalosome that includes PKA regulatory RII subunits and AKAP7, allowing for spatial and functional regulation of cAMP signalling in platelet function [22]. Membrane-associated PDE3A is most likely localised in caveolin-rich plasma membrane lipid rafts, which have been identified in trace amounts in platelets [52]. PDE3A inhibitors, including cilostazol, are used as antiplatelet medications to prevent stroke, treat peripheral artery disease, intermittent claudication and other thrombotic disorders [83,84,85]. However, their use is also associated with unwanted side effects, such as tachycardia, ventricular arrhythmias and hypotension, due to the global inhibition of PDE3 activity.

PDE3A plays a crucial role in maintaining meiotic arrest in oocytes by hydrolysing cAMP, thereby preventing the activation of PKA. A decrease in PDE3A activity allows cAMP levels to rise, leading to PKA activation and the resumption of meiosis. Similarly to platelets, the majority of PDE3A activity in oocytes is attributed to cytosolic isoforms of the enzyme. However, a membrane-associated isoform is also likely present, although its role and significance remain less well characterised [86,87].

While PDE3A is also expressed in various organs and cell types, there remains a significant gap in understanding of its precise subcellular localization within specific cell types. For instance, in the kidney, PDE3A has been detected in juxtaglomerular cells, mesangial cells, distal convoluted tubules and collecting duct cells [88,89,90]. However, detailed knowledge about its localisation within distinct intracellular compartments is still lacking. This contrasts with the cases of some other PDEs, which have already been shown to localise to specific structures. For example, PDE4C in cilia [91], PDE4D in AQP2-bearing vesicles [92] of kidney inner medullary collecting duct cells, PDE3B in cytoplasmic vesicles in distal convoluted tubular cells [93] and PDE1C in the cytoplasm of juxtaglomerular cells [94].

Summarising the available data, it becomes clear that the subcellular localisation of PDE3A largely depends on the structural features of its specific isoforms. PDE3A1, which contains N-terminal transmembrane domains, is usually localised to the membrane fraction of cells. PDE3A2 and PDE3A3 with truncated or absent plasma membrane-association sequences are generally found in soluble cytosolic fractions, with evidence also supporting their nuclear localisation [95]. However, the most critical factor is the molecular context of each PDE3A-containing nanodomain, which ultimately determines the specific role of PDE3A within a distinct subcellular compartment.

## 3. Pharmacologically Targeting PDE3A Activity and Its Protein–Protein Interactions

### 3.1. The PDE3 Family Is an Established Pharmacological Target

The PDE3 family is already targeted for the treatment of cardiovascular diseases with non-selective PDE3A and PDE3B inhibitors, such as milrinone or enoximone [2,7]. Non-selective PDE3 inhibitors increase cAMP and have demonstrated clinical benefits in improving cardiac contractility in late stages of heart failure and lowering blood pressure in pulmonary arterial hypertension (PAH) by promoting vasodilation [55,96,97,98,99,100,101].

The inhibitors do, however, have serious side effects, e.g., thrombocytopenia, a condition with lower platelet counts and an increased risk of bleeding [102]. PDE3 inhibition may cause arrhythmias and long-term treatment of heart failure even increases mortality [50,103]. These cardiac side effects, at least in part, relate to the inhibition of nuclear PDE3, which results in increased HDAC-1 phosphorylation and inhibition of its deacetylase activity. The inhibition derepresses gene transcription, and cardiac myocyte hypertrophic growth (see above). These observations show that more sophisticated strategies are required to target PDE3A and PDE3B individually to increase safety and efficacy.

### 3.2. PDE3A as a Target

Over recent years, specifically PDE3A has emerged as a target for therapeutic intervention in cardiovascular diseases and cancer. Activation of SERCA2a for the treatment of heart failure has been suggested to increase Ca^2+^ reuptake into the SR, improving cardiac contractility [104]. However, clinical trials testing the effect of intracoronary infusion of a SERCA2a cDNA vector did not improve heart failure exacerbations [74,75,76,77,78,79,105]. PDE3A is part of the protein complex organised by AKAP18 that includes SERCA2a, PLN and PKA (Figure 5) [17,37,73,106]. Recently, disruption of the interaction of PDE3A and SERCA2a by targeting the interacting domain with peptides reduced mortality in mice with experimentally induced heart failure [80]. Thus, this strategy of interfering specifically with the PDE3A-SERCA2a interaction may prove to be an alternative for increasing SERCA2a-mediated Ca^2+^ reuptake into the SR.

Gain-of-function PDE3A mutations cause hypertension with brachydactyly (HTNB; Bilingturan syndrome), a rare disease with harmless brachydactyly but progressive, severe hypertension that resembles essential hypertension. Without treatment of their hypertension, the patients die of stroke at around 50 years of age [16,20,34,107,108,109]. Thus, selective PDE3A inhibitors, if specifically delivered to the vascular system, may correct the blood pressure in HTNB patients. Surprisingly, despite their decade-long hypertension HTNB patients do not display the typical hypertension-induced end-organ injuries such as cardiac hypertrophy, heart failure or chronic kidney disease [17,109]. The mechanisms and signalling pathways conferring this protection from hypertension-induced end-organ damage is not known. However, understanding the mechanisms may lead to novel approaches for the prevention and/or treatment of hypertension and hypertension-induced end-organ damage [20].

The Schlafen (SLFN) family comprises more than 10 proteins, most of which have a conserved SLFN domain. The proteins are involved in immune regulation, cell proliferation and differentiation. The interaction of SLFN12 with the catalytic domain of PDE3A activates SLFN12, which cleaves tRNA^Leu^ and induces apoptosis and death of a large variety of cancer cells [110]. Small molecules, molecular glues termed velcrins, link the two proteins and have anti-cancer activity [59,110,111,112,113]. However, in a first human phase I trial the tested compound, Bay 2666605, caused thrombocytopenia despite an only low inhibitory effect on PDE3 activity. Therefore, the trial was terminated but still hints at new options. By medicinal chemistry, it might be possible to redesign the molecular glues.

### 3.3. Targeting PDE3A with Pharmacological Agents

As suggested by the findings outlined in Section 3.2, strategies for selective inhibition or activation of PDE3A would be most useful not only as molecular tools for studying PDE3A functions but prospectively also for therapeutic purposes. While with velcrins (see above), first PDE3A-selective small molecules have been identified, no selective PDE3A inhibitors are available. Available inhibitors non-selectively also inhibit PDE3B [114]. All new pharmacological approaches for inhibiting PDE3A will need to avoid PDE3B inhibition and ideally will specifically inhibit the three PDE3A isoforms individually [114]. The isoforms only differ at their N termini, which direct location. Therefore, approaches targeting the N termini and thus location may be feasible [31,47,114,115]. Such an approach will circumvent the drawback that the catalytic domains cannot be selectively inhibited because they are identical, and most likely will also avoid cross-reactivity with PDE3B because PDE3A and PDE3B display only 35% sequence identity between their N termini, while their catalytic domains share 64% sequence identity (see Section 2.1; Figure 3).

**Figure 6:**
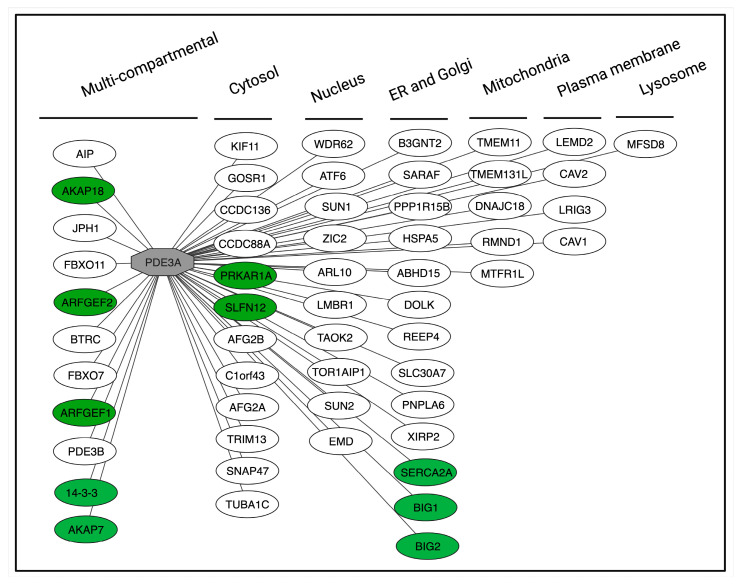
Protein interactions of human PDE3A and their subcellular localisations. PDE3A (grey node) serves as the central hub in the network. The interactions are categorised based on their subcellular localisation. Green nodes represent proteins that physically interact with PDE3A, while white nodes indicate interactions identified through co-immunoprecipitation assays. The figure was adapted from the InAct molecular interaction database (https://www.ebi.ac.uk/intact/home) visualised in Cytoscape Version: 3.10.3 [118].

Due to their specificity, protein–protein interactions are ideal targets [116]. The interaction of PDE3A with SLFN12 and with SERCA2a provide two examples, where increasing the interaction (with SLFN12) or disrupting the interaction (with SERCA2a) has functional consequences (see above). In addition to SLFN12 and SERCA2a, PDE3A interacts with a variety of further proteins in various cellular compartments. The hitherto identified interactions relate to functional as well as physical interactions (Figure 6). For example, the knockdown of PDE3A in HeLa cells decreased the membrane association of guanine nucleotide exchange factor (GEF) for ADP-ribosylation factors (ARFs) 1, ARFGEF1 and ARFGEF2. Whether this functional interaction links to a physical interaction between PDE3A and the two ARFGEFs is not known [38]. The βAR agonist isoproterenol induces PKA phosphorylation of PDE3A1 at S312 and binding of the adapter protein 14-3-3 [31]. Activation of PKC with PMA leads to phosphorylation of PDE3A2 at S428 and also 14-3-3 binding [31,32,114]. Also, physical interactions of PDE3A in HeLa cells with 14-3-3 proteins and protein phosphatase (PP) 2A have been revealed by proteomics [117]. The interactions of PDE3A with 14-3-3 and SERCA2a have been mapped [31]. The direct binding of 14-3-3 to PDE3A shields the phosphorylated sites [31]; in platelets, activated PKC phosphorylates PDE3A at Ser438, Ser465 and Ser492 and enhances 14-3-3 binding, which is associated with increased PDE3A activity [30,32]. Using human cardiac tissue, precipitation experiments showed that PDE3A is part of the AKAP18-based complex comprising PKA, PLN and SERCA2a [37]. Later studies revealed that the interaction is direct and disruption with peptides causes SERCA2 activation [80].

Analysis using the InAct molecular interaction database (https://www.ebi.ac.uk/intact/home) shows various further PDE3A interactions (Figure 6). Mapping and understanding the functions of all PDE3A interactions will provide detailed insight in PDE3A functions and will most likely hint to disease-relevant interactions for modulation and open new avenues towards therapeutic concepts in various diseases.

## 4. Conclusions and Future Directions

PDE3A’s capacity to compartmentalise cAMP by modulating its levels in defined cellular locations provides a sophisticated method of controlling physiological processes. The improved understanding of PDE3A biology has contributed to shaping the concept of cAMP signalling compartments. PDE3A together with PDE3B even constitutes an established therapeutic target.

However, much about the role of PDE3A in cellular signalling and how it functions at the molecular level is unknown. The functions of the three PDE3A isoforms are ill defined, largely due to the lack of isoform-selective tools such as specific antibodies and pharmacological agents. How location of the individual PDE3A isoforms is achieved is unclear, e.g., PDE3A1 is directed to membranes by its two hydrophobic membrane-targeting domains but it is also found in the cytosol; likewise, how PDE3A2 reaches the nucleus is unknown. The protein interactions PDE3A engages in are mostly without ascribed function.

While selective modulators are limited, precise molecular biology tools could be utilised for functional studies. The PDE3A gene could be edited or deleted in part or full using CRISPR-Cas9 for modulating expression and/or activity in a temporally controllable and reversible manner [119].

Only the 3D structure of the catalytic domain of PDE3A is known [113], showing that attempts for full structure elucidation have failed so far. Many protein structures can be predicted with a high degree of probability using AlphaFold 3 [120,121]. However, AlphaFold 3 does not make high confidence structure prediction for the region N-terminal of the catalytic domain. PDE3A is active as a homodimer/oligomer [113,122], the N-terminus inhibits the catalytic domain [17] and PDE3A forms complexes with other proteins [6,123]. In order to elucidate the full-length 3D structure of all PDE3A isoforms these observations would need to be integrated in future experimental structure analyses and in improved AI-based structure predictions.

Elucidation of the structures of the PDE3A1-3 isoforms will not only provide a detailed understanding of the regulation of their activity but is also a prerequisite for rational design or virtual screening to identify not only PDE3A-selective but PDE3A1-, A2- and A3-specific modulators. AI already offers valuable tools and they will be continuously improved. In view of the huge chemical structure space with an estimated >10^60^ molecules, as well as a development time of often 10 years with >1 billion euros in development costs per drug, AI approaches have great potential to save costs and time in drug development. AI has already supported all steps from the identification of a pharmacological target, virtual screening and substance optimisation with regard to efficacy and pharmacological properties [124,125]. AI can create so-called digital twins, in silico replicates, of patients and thus test and optimise therapeutic approaches and reduce risks for patients before they are actually treated [126]. Thus, in the light of the fragmentary knowledge on PDE3A, AI-based approaches will undoubtedly assist in elucidating structure and function as well as in finding selective pharmacological modulators of PDE3A isoforms.

Understanding the function of PDE3A in detail and the availability of selective modulators will clarify the value of PDE3A as a drug target. PDE3A isoforms are almost ubiquitously expressed. Since efficient disease treatment with few or no side effects requires precise targeting of only the relevant tissues, cells and cellular compartments, strategies for directed delivery of any pharmacological agent are needed. An example is given by *peptides* that were transported from lung to heart by nano-in-micro technology (LungToHeartNiM technology) after inhalation and promoted heart recovery in a pig heart failure model [127].

In conclusion, a better understanding of the functions of PDE3A, its isoforms and their interactions in combination with the development of selective pharmacological modulators will not only define its functions but will also unfold the full potential of PDE3A as a therapeutic target and will eventually lead to innovative approaches for the treatment of diseases with an unmet medical need, such as heart failure or hypertension.

## Figures and Tables

**Table 1:** Substrate specificities of PDE families 1–11. PDEs hydrolyse cAMP, cGMP or both (dual-specific).

PDE Family	Hydrolyses cAMP	Hydrolyses cGMP
PDE1	√	√
PDE2	√	√
PDE3	√	√
PDE4	√	×
PDE5	×	√
PDE6	×	√
PDE7	√	×
PDE8	√	×
PDE9	×	√
PDE10	√	√
PDE11	√	√

**Table 3:** PDE3B: Expression pattern, cellular location and key functions.

Cell Type	Expression	Key Functions	Subcellular Localisation	Reference
Adipocytes	High expression in white and brown adipose tissue	Regulates lipolysis by hydrolysing cAMP, affecting hormone-sensitive lipase activity	Caveolae, endoplasmic reticulum	[42,43]
Hepatocytes	Expressed in liver cells	Regulates glucose and lipid metabolism; deficiency leads to gluconeogenesis and lipid accumulation	Caveolae, smooth ER	[44]
Pancreatic β-cells	Moderate	Modulates insulin secretion by regulating cAMP involved in granule exocytosis	Plasma membrane, insulin granules	[45]
Atrial endothelial cells (AECs)	Highly expressed	Regulating AECs adhesion, spreading and tubular formation; vital for angiogenesis	Plasma membrane	[46]

**Table 4:** PDE3A: Expression pattern, cellular location and key functions.

Cell Type	Expression	Key Functions	Subcellular Localisation	References
Vascular smooth muscle cells	High	Regulates vascular tone; inhibition causes vasodilation and lowers blood pressure	Plasma membrane, cytosol	[47,48,49]
Cardiomyocytes	High	Modulates cardiac contractility and cAMP signalling	SR, cytosol, nucleus	[47,50,51]
Platelets	Moderate	Inhibits aggregation via cAMP signalling	Plasma membrane, cytosol	[52]
Human aortic endothelial cells (HAECs)	Low to Moderate	Regulates endothelial barrier and angiogenesis	Plasma membrane, cytosol	[53]
Oocytes	High	Maintains meiotic arrest; inhibition resumes meiosis	Cytoplasm near plasma membrane, perinuclear	[54]
Pulmonary arterial cells	Moderate to High	Involved in vasodilation; target in pulmonary hypertension	Cytoplasm, membrane-associated	[48,55,56]
T-lymphocytes	Low	Possible modulation of cAMP-mediated immune responses	Not well defined, likely cytosolic	[47,57]
Cancer stem cells (breast cancer), intestinal cancer cells, myxoid liposarccoma (SA4, GOT3), cervical cancer (HeLa cells)	Aberrant/High	Promotes proliferation and survival	Nuclear and cytoplasmic (context-dependent)	[2,7,58,59,60,61,62,63]
Brain (cerebelum, cortex, callosum)	Moderately	Neuronal signalling, protection against neural damage	Plasma membrane, cytosol	[64,65,66]

## Abbreviations

The following abbreviations are used in this manuscript:

PDE3A	Phosphodiesterase 3A
cAMP	Cyclic adenosine monophosphate
cNT	Cyclic nucleotides

## References

[1] Anton S.E., Kayser C., Maiellaro I., Nemec K., Moller J., Koschinski A., Zaccolo M., Annibale P., Falcke M., Lohse M.J. (2022). Receptor-associated independent cAMP nanodomains mediate spatiotemporal specificity of GPCR signaling. Cell.

[2] Baillie G.S., Tejeda G.S., Kelly M.P. (2019). Therapeutic targeting of 3′,5′-cyclic nucleotide phosphodiesterases: Inhibition and beyond. Nat. Rev. Drug Discov..

[3] Bock A., Annibale P., Konrad C., Hannawacker A., Anton S.E., Maiellaro I., Zabel U., Sivaramakrishnan S., Falcke M., Lohse M.J. (2020). Optical Mapping of cAMP Signaling at the Nanometer Scale. Cell.

[4] Zaccolo M., Zerio A., Lobo M.J. (2021). Subcellular Organization of the cAMP Signaling Pathway. Pharmacol. Rev..

[5] Klussmann E. (2016). Protein-protein interactions of PDE4 family members–Functions, interactions and therapeutic value. Cell Signal.

[6] Fu Q., Wang Y., Yan C., Xiang Y.K. (2024). Phosphodiesterase in heart and vessels: From physiology to diseases. Physiol. Rev..

[7] Kelly M.P., Nikolaev V.O., Gobejishvili L., Lugnier C., Hesslinger C., Nickolaus P., Kass D.A., Pereira de Vasconcelos W., Fischmeister R., Brocke S. (2025). Cyclic nucleotide phosphodiesterases as drug targets. Pharmacol. Rev..

[8] Dema A., Perets E., Schulz M.S., Deak V.A., Klussmann E. (2015). Pharmacological targeting of AKAP-directed compartmentalized cAMP signalling. Cell Signal.

[9] Bucko P.J., Scott J.D. (2020). Drugs that Regulate Local Cell Signaling: AKAP Targeting as a Therapeutic Option. Annu. Rev. Pharmacol. Toxicol..

[10] Sholokh A., Klussmann E. (2021). Local cyclic adenosine monophosphate signalling cascades-Roles and targets in chronic kidney disease. Acta Physiol..

[11] Subramanian H., Nikolaev V.O. (2023). A-Kinase Anchoring Proteins in Cardiac Myocytes and Their Roles in Regulating Calcium Cycling. Cells.

[12] Beavo J.A., Conti M., Heaslip R.J. (1994). Multiple cyclic nucleotide phosphodiesterases. Mol. Pharmacol..

[13] Bender A.T., Beavo J.A. (2006). Cyclic nucleotide phosphodiesterases: Molecular regulation to clinical use. Pharmacol. Rev..

[14] Conti M., Beavo J. (2007). Biochemistry and physiology of cyclic nucleotide phosphodiesterases: Essential components in cyclic nucleotide signaling. Annu. Rev. Biochem..

[15] Francis S.H., Blount M.A., Corbin J.D. (2011). Mammalian cyclic nucleotide phosphodiesterases: Molecular mechanisms and physiological functions. Physiol. Rev..

[16] Ercu M., Marko L., Schachterle C., Tsvetkov D., Cui Y., Maghsodi S., Bartolomaeus T.U.P., Maass P.G., Zuhlke K., Gregersen N. (2020). Phosphodiesterase 3A and Arterial Hypertension. Circulation.

[17] Ercu M., Mucke M.B., Pallien T., Marko L., Sholokh A., Schachterle C., Aydin A., Kidd A., Walter S., Esmati Y. (2022). Mutant Phosphodiesterase 3A Protects From Hypertension-Induced Cardiac Damage. Circulation.

[18] Trawally M. (2023). Beyond the heart—Exploring the therapeutic potential of PDE3 inhibitors. J. Res. Pharm..

[19] Corpet F. (1988). Multiple sequence alignment with hierarchical clustering. Nucleic Acids Res..

[20] Ercu M., Walter S., Klussmann E. (2023). Mutations in Phosphodiesterase 3A (PDE3A) Cause Hypertension Without Cardiac Damage. Hypertension.

[21] Hambleton R., Krall J., Tikishvili E., Honeggar M., Ahmad F., Manganiello V.C., Movsesian M.A. (2005). Isoforms of Cyclic Nucleotide Phosphodiesterase PDE3 and Their Contribution to cAMP Hydrolytic Activity in Subcellular Fractions of Human Myocardium. J. Biol. Chem..

[22] Khalil J.S., Law R., Raslan Z., Cheah L.T., Hindle M.S., Aburima A.A., Kearney M.T., Naseem K.M. (2024). Protein Kinase A Regulates Platelet Phosphodiesterase 3A through an A-Kinase Anchoring Protein Dependent Manner. Cells.

[23] Blom N., Gammeltoft S., Brunak S. (1999). Sequence and structure-based prediction of eukaryotic protein phosphorylation sites. J. Mol. Biol..

[24] Wong Y.-H., Lee T.-Y., Liang H.-K., Huang C.-M., Wang T.-Y., Yang Y.-H., Chu C.-H., Huang H.-D., Ko M.-T., Hwang J.-K. (2007). KinasePhos 2.0: A web server for identifying protein kinase-specific phosphorylation sites based on sequences and coupling patterns. Nucleic Acids Res..

[25] Taylor S.S., Soberg K., Kobori E., Wu J., Pautz S., Herberg F.W., Skalhegg B.S. (2022). The Tails of Protein Kinase A. Mol. Pharmacol..

[26] Parnell E., Palmer T.M., Yarwood S.J. (2015). The future of EPAC-targeted therapies: Agonism versus antagonism. Trends Pharmacol. Sci..

[27] Gruscheski L., Brand T. (2021). The Role of POPDC Proteins in Cardiac Pacemaking and Conduction. J. Cardiovasc. Dev. Dis..

[28] Hennis K., Piantoni C., Biel M., Fenske S., Wahl-Schott C. (2024). Pacemaker Channels and the Chronotropic Response in Health and Disease. Circ. Res..

[29] Han S.J., Vaccari S., Nedachi T., Andersen C.B., Kovacina K.S., Roth R.A., Conti M. (2006). Protein kinase B/Akt phosphorylation of PDE3A and its role in mammalian oocyte maturation. EMBO J..

[30] Pozuelo Rubio M., Campbell D.G., Morrice N.A., Mackintosh C. (2005). Phosphodiesterase 3A binds to 14-3-3 proteins in response to PMA-induced phosphorylation of Ser428. Biochem. J..

[31] Hunter R.W., MacKintosh C., Hers I. (2009). Protein Kinase C-mediated Phosphorylation and Activation of PDE3A Regulate cAMP Levels in Human Platelets. J. Biol. Chem..

[32] Vandeput F., Szabo-Fresnais N., Ahmad F., Kho C., Lee A., Krall J., Dunlop A., Hazel M.W., Wohlschlegel J.A., Hajjar R.J. (2013). Selective regulation of cyclic nucleotide phosphodiesterase PDE3A isoforms. Proc. Natl. Acad. Sci. USA.

[33] Zemskov E.A., Zemskova M.A., Wu X., Moreno Caceres S., Caraballo Delgado D., Yegambaram M., Lu Q., Fu P., Wang T., Black S.M. (2024). Novel mechanism of cyclic nucleotide crosstalk mediated by PKG-dependent proteasomal degradation of the Hsp90 client protein phosphodiesterase 3A. J. Biol. Chem..

[34] Maass P.G., Aydin A., Luft F.C., Schachterle C., Weise A., Stricker S., Lindschau C., Vaegler M., Qadri F., Toka H.R. (2015). PDE3A mutations cause autosomal dominant hypertension with brachydactyly. Nat. Genet..

[35] Dessauer C.W. (2009). Adenylyl cyclase—A-kinase anchoring protein complexes: The next dimension in cAMP signaling. Mol. Pharmacol..

[36] Beca S., Ahmad F., Shen W., Liu J., Makary S., Polidovitch N., Sun J., Hockman S., Chung Y.W., Movsesian M. (2013). Phosphodiesterase Type 3A Regulates Basal Myocardial Contractility Through Interacting With Sarcoplasmic Reticulum Calcium ATPase Type 2a Signaling Complexes in Mouse Heart. Circ. Res..

[37] Ahmad F., Shen W., Vandeput F., Szabo-Fresnais N., Krall J., Degerman E., Goetz F., Klussmann E., Movsesian M., Manganiello V. (2015). Regulation of sarcoplasmic reticulum Ca^2+^ ATPase 2 (SERCA2) activity by phosphodiesterase 3A (PDE3A) in human myocardium: Phosphorylation-dependent interaction of PDE3A1 with SERCA2. J. Biol. Chem..

[38] Puxeddu E., Uhart M., Li C.-C., Ahmad F., Pacheco-Rodriguez G., Manganiello V.C., Moss J., Vaughan M. (2009). Interaction of phosphodiesterase 3A with brefeldin A-inhibited guanine nucleotide-exchange proteins BIG1 and BIG2 and effect on ARF1 activity. Proc. Natl. Acad. Sci. USA.

[39] Penmatsa H., Zhang W., Yarlagadda S., Li C., Conoley V.G., Yue J., Bahouth S.W., Buddington R.K., Zhang G., Nelson D.J. (2010). Compartmentalized Cyclic Adenosine 3′,5′-Monophosphate at the Plasma Membrane Clusters PDE3A and Cystic Fibrosis Transmembrane Conductance Regulator into Microdomains. MBoC.

[40] Mika D., Leroy J., Vandecasteele G., Fischmeister R. (2012). PDEs create local domains of cAMP signaling. J. Mol. Cell. Cardiol..

[41] Subramaniam G., Schleicher K., Kovanich D., Zerio A., Folkmanaite M., Chao Y.C., Surdo N.C., Koschinski A., Hu J., Scholten A. (2023). Integrated Proteomics Unveils Nuclear PDE3A2 as a Regulator of Cardiac Myocyte Hypertrophy. Circ. Res..

[42] Ahmad F., Lindh R., Tang Y., Ruishalme I., Öst A., Sahachartsiri B., Strålfors P., Degerman E., Manganiello V.C. (2009). Differential regulation of adipocyte PDE3B in distinct membrane compartments by insulin and the β3-adrenergic receptor agonist CL316243: Effects of caveolin-1 knockdown on formation/maintenance of macromolecular signalling complexes. Biochem. J..

[43] Rondinone C.M., Carvalho E., Rahn T., Manganiello V.C., Degerman E., Smith U.P. (2000). Phosphorylation of PDE3B by Phosphatidylinositol 3-Kinase Associated with the Insulin Receptor. J. Biol. Chem..

[44] Berger K., Lindh R., Wierup N., Zmuda-Trzebiatowska E., Lindqvist A., Manganiello V.C., Degerman E. (2009). Phosphodiesterase 3B Is Localized in Caveolae and Smooth ER in Mouse Hepatocytes and Is Important in the Regulation of Glucose and Lipid Metabolism. PLoS ONE.

[45] Degerman E., Ahmad F., Chung Y.W., Guirguis E., Omar B., Stenson L., Manganiello V. (2011). From PDE3B to the regulation of energy homeostasis. Curr. Opin. Pharmacol..

[46] Wilson L.S., Baillie G.S., Pritchard L.M., Umana B., Terrin A., Zaccolo M., Houslay M.D., Maurice D.H. (2011). A Phosphodiesterase 3B-based Signaling Complex Integrates Exchange Protein Activated by cAMP 1 and Phosphatidylinositol 3-Kinase Signals in Human Arterial Endothelial Cells. J. Biol. Chem..

[47] Maurice D.H., Ke H., Ahmad F., Wang Y., Chung J., Manganiello V.C. (2014). Advances in targeting cyclic nucleotide phosphodiesterases. Nat. Rev. Drug Discov..

[48] Liu H., Maurice D.H. (1998). Expression of cyclic GMP-inhibited phosphodiesterases 3A and 3B (PDE3A and PDE3B) in rat tissues: Differential subcellular localization and regulated expression by cyclic AMP. Br. J. Pharmacol..

[49] Omori K., Kotera J. (2007). Overview of PDEs and Their Regulation. Circ. Res..

[50] Movsesian M. (2016). Novel approaches to targeting PDE3 in cardiovascular disease. Pharmacol. Ther..

[51] Movsesian M.A., Bristow M.R. (2005). Alterations in cAMP-Mediated Signaling and Their Role in the Pathophysiology of Dilated Cardiomyopathy. Current Topics in Developmental Biology.

[52] Belleville-Rolland T., Leuci A., Mansour A., Decouture B., Martin F., Poirault-Chassac S., Rouaud M., Guerineau H., Dizier B., Pidard D. (2021). Role of Membrane Lipid Rafts in MRP4 (ABCC4) Dependent Regulation of the cAMP Pathway in Blood Platelets. Thromb. Haemost..

[53] Hashimoto A., Tanaka M., Takeda S., Ito H., Nagano K. (2015). Cilostazol Induces PGI2 Production via Activation of the Downstream Epac-1/Rap1 Signaling Cascade to Increase Intracellular Calcium by PLCε and to Activate p44/42 MAPK in Human Aortic Endothelial Cells. PLoS ONE.

[54] Conti M., Andersen C.B., Richard F., Mehats C., Chun S.-Y., Horner K., Jin C., Tsafriri A. (2002). Role of cyclic nucleotide signaling in oocyte maturation. Mol. Cell. Endocrinol..

[55] Francis S.H. (2011). Phosphodiesterases as Drug Targets.

[56] Fujiwara T., Ishii S., Minatsuki S., Hatano M., Takeda N. (2025). Exploring Novel Therapeutics for Pulmonary Arterial Hypertension from the Bench to the Bedside. Int. Heart J..

[57] Ekholm D., Hemmer B., Gao G., Vergelli M., Martin R., Manganiello V. (1997). Differential expression of cyclic nucleotide phosphodiesterase 3 and 4 activities in human T cell clones specific for myelin basic protein. J. Immunol..

[58] Krause P.N., McGeorge G., McPeek J.L., Khalid S., Nelin L.D., Liu Y., Chen B. (2024). Pde3a and Pde3b regulation of murine pulmonary artery smooth muscle cell growth and metabolism. Physiol. Rep..

[59] Greulich H., Kaplan B., Mertins P., Chen T.-H., Tanaka K.E., Yun C.-H., Zhang X., Lee S.-H., Cho J., Ambrogio L. (2012). Functional analysis of receptor tyrosine kinase mutations in lung cancer identifies oncogenic extracellular domain mutations of ERBB2. Proc. Natl. Acad. Sci. USA.

[60] Hao N., Shen W., Du R., Jiang S., Zhu J., Chen Y., Huang C., Shi Y., Xiang R., Luo Y. (2020). Phosphodiesterase 3A Represents a Therapeutic Target that Drives Stem Cell–like Property and Metastasis in Breast Cancer. Mol. Cancer Ther..

[61] Vandenberghe P., Hagué P., Hockman S.C., Manganiello V.C., Demetter P., Erneux C., Vanderwinden J.-M. (2017). Phosphodiesterase 3A: A new player in development of interstitial cells of Cajal and a prospective target in gastrointestinal stromal tumors (GIST). Oncotarget.

[62] Toivanen K., Kilpinen S., Ojala K., Merikoski N., Salmikangas S., Sampo M., Böhling T., Sihto H. (2023). PDE3A Is a Highly Expressed Therapy Target in Myxoid Liposarcoma. Cancers.

[63] Nazir M., Senkowski W., Nyberg F., Blom K., Edqvist P.-H., Jarvius M., Andersson C., Gustafsson M.G., Nygren P., Larsson R. (2017). Targeting tumor cells based on Phosphodiesterase 3A expression. Exp. Cell Res..

[64] Argyrousi E.K., Heckman P.R.A., Prickaerts J. (2020). Role of cyclic nucleotides and their downstream signaling cascades in memory function: Being at the right time at the right spot. Neurosci. Biobehav. Rev..

[65] Reinhardt R.R., Bondy C.A. (1996). Differential cellular pattern of gene expression for two distinct cGMP-inhibited cyclic nucleotide phosphodiesterases in developing and mature rat brain. Neuroscience.

[66] Mitome-Mishima Y., Miyamoto N., Tanaka R., Oishi H., Arai H., Hattori N., Urabe T. (2013). Differences in phosphodiesterase 3A and 3B expression after ischemic insult. Neurosci. Res..

[67] Weiss S., Oz S., Benmocha A., Dascal N. (2013). Regulation of cardiac L-type Ca^2+^ channel Ca_V_1.2 via the beta-adrenergic-cAMP-protein kinase A pathway: Old dogmas, advances, and new uncertainties. Circ. Res..

[68] Pallien T., Klussmann E. (2020). New aspects in cardiac L-type Ca^2+^ channel regulation. Biochem. Soc. Trans..

[69] Oz S., Keren-Raifman T., Sharon T., Subramaniam S., Pallien T., Katz M., Tsemakhovich V., Sholokh A., Watad B., Tripathy D.R. (2024). Tripartite interactions of PKA catalytic subunit and C-terminal domains of cardiac Ca^2+^ channel may modulate its beta-adrenergic regulation. BMC Biol..

[70] Wu H., Lee J., Vincent L.G., Wang Q., Gu M., Lan F., Churko J.M., Sallam K.I., Matsa E., Sharma A. (2015). Epigenetic Regulation of Phosphodiesterases 2A and 3A Underlies Compromised beta-Adrenergic Signaling in an iPSC Model of Dilated Cardiomyopathy. Cell Stem Cell.

[71] Zhao C.Y., Greenstein J.L., Winslow R.L. (2015). Interaction between phosphodiesterases in the regulation of the cardiac beta-adrenergic pathway. J. Mol. Cell. Cardiol..

[72] Pavlaki N., De Jong K.A., Geertz B., Nikolaev V.O., Froese A. (2021). Cardiac Hypertrophy Changes Compartmentation of cAMP in Non-Raft Membrane Microdomains. Cells.

[73] Lygren B., Carlson C.R., Santamaria K., Lissandron V., McSorley T., Litzenberg J., Lorenz D., Wiesner B., Rosenthal W., Zaccolo M. (2007). AKAP complex regulates Ca^2+^ re-uptake into heart sarcoplasmic reticulum. EMBO Rep..

[74] Gorski P.A., Ceholski D.K., Young H.S., Krebs J. (2017). Structure-Function Relationship of the SERCA Pump and Its Regulation by Phospholamban and Sarcolipin. Membrane Dynamics and Calcium Signaling.

[75] Hamm N.C., Stammers A.N., Susser S.E., Hlynsky M.W., Kimber D.E., Kehler D.S., Duhamel T.A., Chakraborti S., Dhalla N.S. (2016). Regulation of Cardiac Sarco(endo)plasmic Reticulum Calcium-ATPases (SERCA2a) in Response to Exercise. Regulation of Ca^2+^-ATPases, V-ATPases and F-ATPases.

[76] Kiess T.-O., Kockskämper J. (2019). SERCA Activity Controls the Systolic Calcium Increase in the Nucleus of Cardiac Myocytes. Front. Physiol..

[77] Lefkimmiatis K., Zaccolo M. (2014). cAMP signaling in subcellular compartments. Pharmacol. Ther..

[78] Nemirovskaya T.L., Sharlo K.A. (2022). Roles of ATP and SERCA in the Regulation of Calcium Turnover in Unloaded Skeletal Muscles: Current View and Future Directions. Int. J. Mol. Sci..

[79] Xu H., Van Remmen H. (2021). The SarcoEndoplasmic Reticulum Calcium ATPase (SERCA) pump: A potential target for intervention in aging and skeletal muscle pathologies. Skelet. Muscle.

[80] Skogestad J., Albert I., Hougen K., Lothe G.B., Lunde M., Eken O.S., Veras I., Huynh N.T.T., Borstad M., Marshall S. (2023). Disruption of Phosphodiesterase 3A Binding to SERCA2 Increases SERCA2 Activity and Reduces Mortality in Mice With Chronic Heart Failure. Circulation.

[81] Touyz R.M., Alves-Lopes R., Rios F.J., Camargo L.L., Anagnostopoulou A., Arner A., Montezano A.C. (2018). Vascular smooth muscle contraction in hypertension. Cardiovasc. Res..

[82] Begum N., Hockman S., Manganiello V.C. (2011). Phosphodiesterase 3A (PDE3A) Deletion Suppresses Proliferation of Cultured Murine Vascular Smooth Muscle Cells (VSMCs) via Inhibition of Mitogen-activated Protein Kinase (MAPK) Signaling and Alterations in Critical Cell Cycle Regulatory Proteins. J. Biol. Chem..

[83] Kalantzi K., Tentolouris N., Melidonis A.J., Papadaki S., Peroulis M., Amantos K.A., Andreopoulos G., Bellos G.I., Boutel D., Bristianou M. (2021). Efficacy and Safety of Adjunctive Cilostazol to Clopidogrel-Treated Diabetic Patients with Symptomatic Lower Extremity Artery Disease in the Prevention of Ischemic Vascular Events. J. Am. Heart Assoc..

[84] Suarez Ferreira S.P., Hall R., Majumdar M., Goudot G., Jessula S., Feldman Z.M., Bellomo T., Lee I., Owolabi L., Kirshkaln-Leahy A. (2023). Effect of Cilostazol in Platelet Inhibition in Patients with Peripheral Artery Disease. J. Vasc. Surg..

[85] Sohn M., Lim S. (2024). The Role of Cilostazol, a Phosphodiesterase-3 Inhibitor, in the Development of Atherosclerosis and Vascular Biology: A Review with Meta-Analysis. Int. J. Mol. Sci..

[86] Begum N., Shen W., Manganiello V. (2011). Role of PDE3A in regulation of cell cycle progression in mouse vascular smooth muscle cells and oocytes: Implications in cardiovascular diseases and infertility. Curr. Opin. Pharmacol..

[87] Shitsukawa K., Andersen C.B., Richard F.J., Horner A.K., Wiersma A., van Duin M., Conti M. (2001). Cloning and characterization of the cyclic guanosine monophosphate-inhibited phosphodiesterase PDE3A expressed in mouse oocyte. Biol. Reprod..

[88] Friis U.G., Madsen K., Stubbe J., Hansen P.B., Svenningsen P., Bie P., Skott O., Jensen B.L. (2013). Regulation of renin secretion by renal juxtaglomerular cells. Pflug. Arch..

[89] Dousa T.P. (1999). Cyclic-3′,5′-nucleotide phosphodiesterase isozymes in cell biology and pathophysiology of the kidney. Kidney Int..

[90] Zhu Y., Yao J., Meng Y., Kasai A., Hiramatsu N., Hayakawa K., Miida T., Takeda M., Okada M., Kitamura M. (2006). Profiling of functional phosphodiesterase in mesangial cells using a CRE-SEAP-based reporting system. Br. J. Pharmacol..

[91] Torres V.E., Harris P.C. (2014). Strategies targeting cAMP signaling in the treatment of polycystic kidney disease. J. Am. Soc. Nephrol..

[92] Stefan E., Wiesner B., Baillie G.S., Mollajew R., Henn V., Lorenz D., Furkert J., Santamaria K., Nedvetsky P., Hundsrucker C. (2007). Compartmentalization of cAMP-dependent signaling by phosphodiesterase-4D is involved in the regulation of vasopressin-mediated water reabsorption in renal principal cells. J. Am. Soc. Nephrol..

[93] Wang X., Ward C.J., Harris P.C., Torres V.E. (2010). Cyclic nucleotide signaling in polycystic kidney disease. Kidney Int..

[94] Ortiz-Capisano M.C., Liao T.D., Ortiz P.A., Beierwaltes W.H. (2009). Calcium-dependent phosphodiesterase 1C inhibits renin release from isolated juxtaglomerular cells. Am. J. Physiol. Regul. Integr. Comp. Physiol..

[95] Shakur Y., Takeda K., Kenan Y., Yu Z.X., Rena G., Brandt D., Houslay M.D., Degerman E., Ferrans V.J., Manganiello V.C. (2000). Membrane localization of cyclic nucleotide phosphodiesterase 3 (PDE3). Two N-terminal domains are required for the efficient targeting to, and association of, PDE3 with endoplasmic reticulum. J. Biol. Chem..

[96] Chen Z., Zhao K., Xiao C., He Z., Liu S., Wu X., Shi S., Guo Y. (2022). Phosphodiesterase inhibitor for heart failure with preserved ejection fraction: A systematic review and meta-analysis. Saudi. Pharm. J..

[97] Feneck R. (2008). Phosphodiesterase inhibitors and the cardiovascular system. Contin. Educ. Anaesth. Crit. Care Pain..

[98] Kamel R., Leroy J., Vandecasteele G., Fischmeister R. (2023). Cyclic nucleotide phosphodiesterases as therapeutic targets in cardiac hypertrophy and heart failure. Nat. Rev. Cardiol..

[99] Kherallah R.Y., Khawaja M., Olson M., Angiolillo D., Birnbaum Y. (2022). Cilostazol: A Review of Basic Mechanisms and Clinical Uses. Cardiovasc. Drugs Ther..

[100] Mokry J., Mokra D. (2013). Immunological aspects of phosphodiesterase inhibition in the respiratory system. Respir. Physiol. Neurobiol..

[101] Sala V., Margaria J.P., Murabito A., Morello F., Ghigo A., Hirsch E. (2017). Therapeutic Targeting of PDEs and PI3K in Heart Failure with Preserved Ejection Fraction (HFpEF). Curr. Heart Fail. Rep..

[102] Papadopoulos K.P., McKean M., Goldoni S., Genvresse I., Garrido M.F., Li R., Wilkinson G., Kneip C., Yap T.A. (2024). First-in-Human Dose-Escalation Study of the First-in-Class PDE3A-SLFN12 Complex Inducer BAY 2666605 in Patients with Advanced Solid Tumors Coexpressing SLFN12 and PDE3A. Clin. Cancer Res..

[103] Hoffman T.M. (2018). Phosphodiesterase Inhibitors. Heart Failure in the Child and Young Adult.

[104] Ahmad T., Miller P.E., McCullough M., Desai N.R., Riello R., Psotka M., Bohm M., Allen L.A., Teerlink J.R., Rosano G.M.C. (2019). Why has positive inotropy failed in chronic heart failure? Lessons from prior inotrope trials. Eur. J. Heart Fail..

[105] Greenberg B., Butler J., Felker G.M., Ponikowski P., Voors A.A., Desai A.S., Barnard D., Bouchard A., Jaski B., Lyon A.R. (2016). Calcium upregulation by percutaneous administration of gene therapy in patients with cardiac disease (CUPID 2): A randomised, multinational, double-blind, placebo-controlled, phase 2b trial. Lancet.

[106] McSorley T., Stefan E., Henn V., Wiesner B., Baillie G.S., Houslay M.D., Rosenthal W., Klussmann E. (2006). Spatial organisation of AKAP18 and PDE4 isoforms in renal collecting duct principal cells. Eur. J. Cell Biol..

[107] Luft F.C. (2024). Personal Genetic-Hypertension Odyssey from Phenotypes to Genotypes and Targets. Hypertension.

[108] Schuster H., Wienker T.E., Bahring S., Bilginturan N., Toka H.R., Neitzel H., Jeschke E., Toka O., Gilbert D., Lowe A. (1996). Severe autosomal dominant hypertension and brachydactyly in a unique Turkish kindred maps to human chromosome 12. Nat. Genet..

[109] Sholokh A., Walter S., Marko L., McMurray B.J., Sunaga-Franze D.Y., Xu M., Zuhlke K., Russwurm M., Bartolomaeus T.U.P., Langanki R. (2023). Mutant phosphodiesterase 3A protects the kidney from hypertension-induced damage. Kidney Int..

[110] Ai Y., He H., Chen P., Yan B., Zhang W., Ding Z., Li D., Chen J., Ma Y., Cao Y. (2020). An alkaloid initiates phosphodiesterase 3A-schlafen 12 dependent apoptosis without affecting the phosphodiesterase activity. Nat. Commun..

[111] Aquilanti E., Goldoni S., Baker A., Kotynkova K., Andersen S., Bozinov V., Gao G.F., Cherniack A.D., Lange M., Lesche R. (2024). Velcrin molecular glues induce apoptosis in glioblastomas with high *PDE*_3_*A* and SLFN_12_ expression. Neuro-Oncol. Adv..

[112] Garvie C.W., Wu X., Papanastasiou M., Lee S., Fuller J., Schnitzler G.R., Horner S.W., Baker A., Zhang T., Mullahoo J.P. (2021). Structure of PDE3A-SLFN12 complex reveals requirements for activation of SLFN12 RNase. Nat. Commun..

[113] Lee S., Hoyt S., Wu X., Garvie C., McGaunn J., Shekhar M., Tötzl M., Rees M.G., Cherniack A.D., Meyerson M. (2023). Velcrin-induced selective cleavage of tRNALeu(TAA) by SLFN12 causes cancer cell death. Nat. Chem. Biol..

[114] Wechsler J., Choi Y.H., Krall J., Ahmad F., Manganiello V.C., Movsesian M.A. (2002). Isoforms of cyclic nucleotide phosphodiesterase PDE3A in cardiac myocytes. J. Biol. Chem..

[115] Zhang W., Colman R.W. (2000). Conserved amino acids in metal-binding motifs of PDE3A are involved in substrate and inhibitor binding. Blood.

[116] Klussmann E., Rosenthal W. (2008). Protein-protein interactions as new drug targets. Preface. Handb. Exp. Pharmacol..

[117] Corradini E., Klaasse G., Leurs U., Heck A.J.R., Martin N.I., Scholten A. (2015). Charting the interactome of PDE3A in human cells using an IBMX based chemical proteomics approach. Mol. BioSyst..

[118] Otasek D., Morris J.H., Bouças J., Pico A.R., Demchak B. (2019). Cytoscape Automation: Empowering workflow-based network analysis. Genome Biol..

[119] Schambach A., Buchholz C.J., Torres-Ruiz R., Cichutek K., Morgan M., Trapani I., Büning H. (2024). A new age of precision gene therapy. Lancet.

[120] Abramson J., Adler J., Dunger J., Evans R., Green T., Pritzel A., Ronneberger O., Willmore L., Ballard A.J., Bambrick J. (2024). Accurate structure prediction of biomolecular interactions with AlphaFold 3. Nature.

[121] Abramson J., Adler J., Dunger J., Evans R., Green T., Pritzel A., Ronneberger O., Willmore L., Ballard A.J., Bambrick J. (2024). Addendum: Accurate structure prediction of biomolecular interactions with AlphaFold 3. Nature.

[122] Kenan Y., Murata T., Shakur Y., Degerman E., Manganiello V.C. (2000). Functions of the N-terminal region of cyclic nucleotide phosphodiesterase 3 (PDE 3) isoforms. J. Biol. Chem..

[123] Ercu M., Klussmann E. (2018). Roles of A-Kinase Anchoring Proteins and Phosphodiesterases in the Cardiovascular System. J. Cardiovasc. Dev. Dis..

[124] Ocana A., Pandiella A., Privat C., Bravo I., Luengo-Oroz M., Amir E., Gyorffy B. (2025). Integrating artificial intelligence in drug discovery and early drug development: A transformative approach. Biomark. Res..

[125] Paul D., Sanap G., Shenoy S., Kalyane D., Kalia K., Tekade R.K. (2021). Artificial intelligence in drug discovery and development. Drug Discov. Today.

[126] Bordukova M., Makarov N., Rodriguez-Esteban R., Schmich F., Menden M.P. (2024). Generative artificial intelligence empowers digital twins in drug discovery and clinical trials. Expert Opin. Drug Discov..

[127] Alogna A., Berboth L., Faragli A., Otvos J., Lo Muzio F.P., di Mauro V., Modica J., Quarta E., Semmler L., Deissler P.M. (2024). Lung-to-Heart Nano-in-Micro Peptide Promotes Cardiac Recovery in a Pig Model of Chronic Heart Failure. J. Am. Coll. Cardiol..

